# 9-(1,1-Dimethyl-3-oxobut­yl)adenine

**DOI:** 10.1107/S1600536810034628

**Published:** 2010-09-04

**Authors:** Dulin Kong, Mingshu Wu, Huiyan Li, Jingya Ma

**Affiliations:** a Key Laboratory of Tropical Medicinal Plant Chemistry of the Ministry of Education, College of Chemistry & Chemical Engineering, Hainan Normal University, Haikou 571158, People’s Republic of China; b Environmental Engineering Department, Environmental Management College of China, Qinhuangdao 066004, People’s Republic of China

## Abstract

The title compound, C_11_H_15_N_5_O, crystallizes with two independent mol­ecules in the asymmetric unit, both of which contain essentially planar imidazole and pyrimidine rings [maximum deviations = 0.002 (2) and 0.026 (2) Å, respectively, for the first mol­ecule, and 0.001 (2) and 0.025 (2) Å for the second]; the dihedral angles between the rings are 2.1 (2) and 1.7 (2)° in the two mol­ecules. The crystal structure is stabilized by inter­molecular N—H⋯N hydrogen bonds, defining chains along *a*, which are further linked by weak inter­molecular π–π contacts [centroid centroid distance = 3.7989 (16) Å] into planes parallel to (01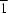).

## Related literature

For the synthesis of the title compound, see: Jiang & Tang (1995 ▶). For the biological activity of related compounds, see: Jeffery *et al.* (2000 ▶); Bayes *et al.* (2003 ▶). For related structures, see: Bo *et al.* (2006 ▶); Deng *et al.* (1995 ▶); Wei *et al.* (2007 ▶); Yu *et al.* (1990 ▶).
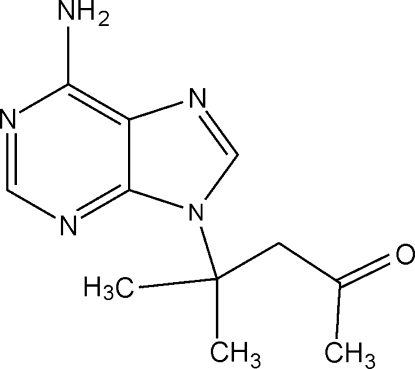


## Experimental

### 

#### Crystal data

C_11_H_15_N_5_O
*M* *_r_* = 233.28
Triclinic, 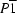

*a* = 8.2565 (8) Å
*b* = 11.2229 (11) Å
*c* = 13.4021 (13) Å
α = 78.421 (1)°
β = 89.551 (2)°
γ = 88.483 (1)°
*V* = 1216.2 (2) Å^3^
*Z* = 4
Mo *K*α radiation
μ = 0.09 mm^−1^
*T* = 298 K
0.48 × 0.47 × 0.14 mm

#### Data collection

Bruker APEXII CCD area-detector diffractometer
Absorption correction: multi-scan (*SADABS*; Bruker, 2005 ▶) *T*_min_ = 0.96, *T*_max_ = 0.99
6288 measured reflections
4208 independent reflections
2538 reflections with *I* > 2σ(*I*)
*R*_int_ = 0.030

#### Refinement

*R*[*F* ^2^ > 2σ(*F* ^2^)] = 0.056
*wR*(*F* ^2^) = 0.162
*S* = 1.06
4208 reflections
313 parameters
H-atom parameters constrained
Δρ_max_ = 0.24 e Å^−3^
Δρ_min_ = −0.22 e Å^−3^

### 

Data collection: *APEX2* (Bruker, 2005 ▶); cell refinement: *SAINT* (Bruker, 2005 ▶); data reduction: *SAINT*; program(s) used to solve structure: *SHELXS97* (Sheldrick, 2008 ▶); program(s) used to refine structure: *SHELXL97* (Sheldrick, 2008 ▶); molecular graphics: *Mercury* (Macrae *et al.*, 2006 ▶); software used to prepare material for publication: *SHELXL97*.

## Supplementary Material

Crystal structure: contains datablocks global, I. DOI: 10.1107/S1600536810034628/bg2361sup1.cif

Structure factors: contains datablocks I. DOI: 10.1107/S1600536810034628/bg2361Isup2.hkl

Additional supplementary materials:  crystallographic information; 3D view; checkCIF report

## Figures and Tables

**Table 1:** Hydrogen-bond geometry (Å, °)

*D*—H⋯*A*	*D*—H	H⋯*A*	*D*⋯*A*	*D*—H⋯*A*
N10—H10*B*⋯N4^i^	0.86	2.28	3.051 (3)	149
N10—H10*A*⋯N2^ii^	0.86	2.23	3.072 (3)	166
N5—H5*B*⋯N9^iii^	0.86	2.16	2.988 (3)	161
N5—H5*A*⋯N7^iv^	0.86	2.20	3.064 (3)	178

Symmetry codes: (i) 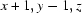; (ii) 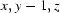; (iii) 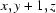; (iv) 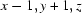.

**Fig. 1.**
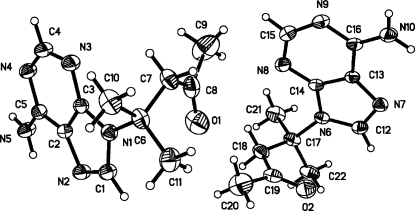
View of the title compound without the hydrogen atom-labeling scheme. Displacement ellipsoids are drawn at the 30% probability level.

**Fig. 2.**
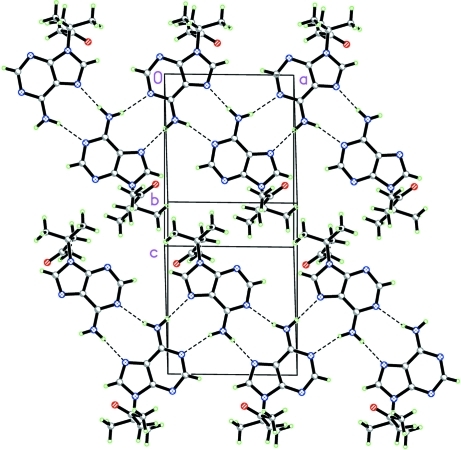
A view of the molecular packing along a axis.

## supplementary crystallographic information

### Comment

Adenine and adenine derivatives have drawn great attention of biochemists and organic synthetic chemists in recent years because of its good active biologic quality. Especially in the respects of antivirus and restraint of cancer cells(Jeffery *et al.*, 2000). They are found in a variety of biologically active molecules(Bayes *et al.*, 2003). The title compound was a new purine alkaloid, isolated from the mycelium of Ganoderma Capense (Lloyd) Teng obtained by submerged fermentation(Yu *et al.*, 1990). The Ganoderma Gapense (Lloyd) Teng is a famous traditional Chinese medicine, and the content of the title compound was very poor, only 8 × 10^-5^ from the corresponding mycelium. Herein, we report the synthesis and crystal structure of the title compound.

The title compound crystallizes with two independent but closely similar molecules per asymmetric unit(Fig 1). Both contain nearly planar imidazole and pyrimidine rings are essentially planar, with maximum deviations of 0.002 (2), 0.026 (2), 0.001 (2) and 0.025 (2) Å, respectively. The dihedral angles between imidazole and pyrimidine rings are 2.14 (20) and 1.74 (20)° respectively. The torsion angles C3—N1—C6—C10, C3—N1—C6—C11, C1—N1—C6—C10, C1—N1—C6—C11 and N1—C6—C7—C8 are 64.0 (4), -176.7 (3), -123.4 (3), -4.0 (4) and -52.1 (4)° respectively. The corresponding values of torsion angles for the second distinct conformer are 64.0 (4), -177.1 (3), -126.0 (3), -7.0 (4) and -56.0 (3)° respectively. The bond angles of C1—N1—C3(105.2 (2)°), C3—N1—C6(127.1 (2)°), C1—N1—C6(127.4 (2)°), C14—N6—C17(126.3 (2)°), C12—N6—C14(105.3 (2)°) and C12—N6—C17(127.9 (2)°) are almost 120°, suggesting the N1 and N6 are *sp*^2^ instead of traditionally *sp*^3^ hybridized with triangular plannar geometry. All bond lengths are normal(Bo *et al.*, 2006).

In the crystal, molecules are linked by intermolecular N–H···N hydrogen-bonds (Table 1) to form an infinite one-dimensional zigzag chain running alomg a (Fig.2), in turn connected by π–π contacts involving the N3—C4, N6—C14 N8—C15 rings, defining two-dimensional layers parallel to (011).

### Experimental

The title molecule was prepared according to literature procedure (Jiang *et al.*, 1995). The compound was dissolved in a minimal amount of DMSO and the solution was then placed in a chamber saturated with dichloromethane at room temperature, covered and allowed to crystallize for two weeks. The resulting pale yellow crystals were collected by filtration, and a suitable crystal was selected for structural determination.

### Refinement

All H atoms were placed in calculated positions, with N–H = 0.86 and C—H = 0.93, 0.96 or 0.97 Å, and included in the final cycles of refinement using a riding model, with *U*_iso_(H) = 1.2*U*_eq_(C).

### Crystal data

**Table:**

C_11_H_15_N_5_O	*Z* = 4
*M**_r_* = 233.28	*F*(000) = 496
Triclinic, *P*1	*D*_x_ = 1.274 Mg m^−^^3^
Hall symbol: -P 1	Mo *K*α radiation, λ = 0.71073 Å
*a* = 8.2565 (8) Å	Cell parameters from 1894 reflections
*b* = 11.2229 (11) Å	θ = 2.7–24.3°
*c* = 13.4021 (13) Å	µ = 0.09 mm^−^^1^
α = 78.421 (1)°	*T* = 298 K
β = 89.551 (2)°	Block, colourless
γ = 88.483 (1)°	0.48 × 0.47 × 0.14 mm
*V* = 1216.2 (2) Å^3^	

### Data collection

**Table:**

Bruker APEXII CCD area-detector diffractometer	4208 independent reflections
Radiation source: fine-focus sealed tube	2538 reflections with *I* > 2σ(*I*)
graphite	*R*_int_ = 0.030
phi and ω scans	θ_max_ = 25.0°, θ_min_ = 1.6°
Absorption correction: multi-scan (*SADABS*; Bruker, 2005)	*h* = −9→9
*T*_min_ = 0.96, *T*_max_ = 0.99	*k* = −13→12
6288 measured reflections	*l* = −15→15

### Refinement

**Table:**

Refinement on *F*^2^	Primary atom site location: structure-invariant direct methods
Least-squares matrix: full	Secondary atom site location: difference Fourier map
*R*[*F*^2^ > 2σ(*F*^2^)] = 0.056	Hydrogen site location: inferred from neighbouring sites
*wR*(*F*^2^) = 0.162	H-atom parameters constrained
*S* = 1.06	*w* = 1/[σ^2^(*F*_o_^2^) + (0.0626*P*)^2^ + 0.3212*P*] where *P* = (*F*_o_^2^ + 2*F*_c_^2^)/3
4208 reflections	(Δ/σ)_max_ < 0.001
313 parameters	Δρ_max_ = 0.24 e Å^−^^3^
0 restraints	Δρ_min_ = −0.22 e Å^−^^3^

### Special details

**Table:**

Geometry. All e.s.d.'s (except the e.s.d. in the dihedral angle between two l.s. planes) are estimated using the full covariance matrix. The cell e.s.d.'s are taken into account individually in the estimation of e.s.d.'s in distances, angles and torsion angles; correlations between e.s.d.'s in cell parameters are only used when they are defined by crystal symmetry. An approximate (isotropic) treatment of cell e.s.d.'s is used for estimating e.s.d.'s involving l.s. planes.
Refinement. Refinement of *F*^2^ against ALL reflections. The weighted *R*-factor *wR* and goodness of fit *S* are based on *F*^2^, conventional *R*-factors *R* are based on *F*, with *F* set to zero for negative *F*^2^. The threshold expression of *F*^2^ > σ(*F*^2^) is used only for calculating *R*-factors(gt) *etc*. and is not relevant to the choice of reflections for refinement. *R*-factors based on *F*^2^ are statistically about twice as large as those based on *F*, and *R*- factors based on ALL data will be even larger.

### Fractional atomic coordinates and isotropic or equivalent isotropic displacement parameters (Å^2^)

**Table:**

	*x*	*y*	*z*	*U*_iso_*/*U*_eq_	
N1	0.2572 (3)	0.8076 (2)	0.01198 (17)	0.0416 (6)	
N2	0.3297 (3)	0.9315 (2)	0.11520 (19)	0.0483 (6)	
N3	−0.0300 (3)	0.7899 (2)	0.0569 (2)	0.0498 (7)	
N4	−0.1029 (3)	0.9008 (2)	0.18776 (19)	0.0480 (7)	
N5	0.0816 (3)	1.0018 (2)	0.26608 (19)	0.0529 (7)	
H5A	0.0059	1.0165	0.3067	0.063*	
H5B	0.1776	1.0275	0.2720	0.063*	
N6	0.7455 (3)	0.2103 (2)	0.49198 (17)	0.0410 (6)	
N7	0.8180 (3)	0.0572 (2)	0.41366 (18)	0.0437 (6)	
N8	0.4600 (3)	0.2330 (2)	0.44317 (19)	0.0493 (7)	
N9	0.3889 (3)	0.0939 (2)	0.33544 (19)	0.0471 (6)	
N10	0.5742 (3)	−0.0341 (2)	0.2781 (2)	0.0542 (7)	
H10A	0.5002	−0.0547	0.2405	0.065*	
H10B	0.6704	−0.0653	0.2779	0.065*	
O1	0.4200 (4)	0.5832 (2)	0.1274 (2)	0.0890 (9)	
O2	0.9215 (3)	0.3952 (2)	0.34758 (19)	0.0747 (7)	
C1	0.3770 (4)	0.8703 (3)	0.0464 (2)	0.0477 (8)	
H1	0.4832	0.8695	0.0229	0.057*	
C2	0.1666 (3)	0.9074 (2)	0.1277 (2)	0.0378 (7)	
C3	0.1211 (3)	0.8310 (2)	0.0644 (2)	0.0386 (7)	
C4	−0.1302 (4)	0.8313 (3)	0.1201 (3)	0.0514 (8)	
H4	−0.2371	0.8082	0.1169	0.062*	
C5	0.0503 (3)	0.9394 (3)	0.1940 (2)	0.0420 (7)	
C6	0.2743 (4)	0.7212 (3)	−0.0589 (2)	0.0478 (8)	
C7	0.2204 (4)	0.5963 (3)	−0.0013 (2)	0.0559 (9)	
H7A	0.1040	0.6009	0.0083	0.067*	
H7B	0.2414	0.5381	−0.0447	0.067*	
C8	0.2954 (5)	0.5463 (3)	0.1002 (3)	0.0608 (9)	
C9	0.2085 (5)	0.4448 (5)	0.1656 (4)	0.1136 (18)	
H9A	0.2738	0.4109	0.2239	0.170*	
H9B	0.1882	0.3830	0.1274	0.170*	
H9C	0.1075	0.4751	0.1878	0.170*	
C10	0.1629 (4)	0.7668 (3)	−0.1489 (2)	0.0661 (10)	
H10C	0.0531	0.7699	−0.1253	0.099*	
H10D	0.1713	0.7126	−0.1959	0.099*	
H10E	0.1939	0.8467	−0.1826	0.099*	
C11	0.4479 (4)	0.7178 (3)	−0.0957 (3)	0.0684 (10)	
H11A	0.4784	0.7983	−0.1283	0.103*	
H11B	0.4575	0.6646	−0.1434	0.103*	
H11C	0.5179	0.6885	−0.0387	0.103*	
C12	0.8644 (4)	0.1328 (3)	0.4701 (2)	0.0452 (7)	
H12	0.9701	0.1334	0.4934	0.054*	
C13	0.6556 (3)	0.0876 (2)	0.3974 (2)	0.0371 (7)	
C14	0.6081 (3)	0.1810 (2)	0.4449 (2)	0.0383 (7)	
C15	0.3592 (4)	0.1818 (3)	0.3882 (2)	0.0510 (8)	
H15	0.2527	0.2113	0.3860	0.061*	
C16	0.5397 (3)	0.0464 (3)	0.3364 (2)	0.0403 (7)	
C17	0.7629 (4)	0.3162 (3)	0.5422 (2)	0.0485 (8)	
C18	0.7157 (4)	0.4315 (3)	0.4650 (2)	0.0544 (9)	
H18A	0.6011	0.4286	0.4506	0.065*	
H18B	0.7303	0.5007	0.4970	0.065*	
C19	0.8052 (4)	0.4549 (3)	0.3652 (3)	0.0539 (8)	
C20	0.7440 (5)	0.5588 (4)	0.2860 (3)	0.0932 (14)	
H20A	0.8196	0.5739	0.2304	0.140*	
H20B	0.7319	0.6301	0.3150	0.140*	
H20C	0.6409	0.5393	0.2616	0.140*	
C21	0.6479 (5)	0.3011 (3)	0.6328 (3)	0.0689 (10)	
H21A	0.5399	0.2908	0.6106	0.103*	
H21B	0.6503	0.3722	0.6624	0.103*	
H21C	0.6810	0.2309	0.6826	0.103*	
C22	0.9356 (4)	0.3201 (3)	0.5801 (3)	0.0658 (10)	
H22A	0.9617	0.2457	0.6271	0.099*	
H22B	0.9449	0.3875	0.6137	0.099*	
H22C	1.0091	0.3296	0.5234	0.099*	

### Atomic displacement parameters (Å^2^)

**Table:**

	*U*^11^	*U*^22^	*U*^33^	*U*^12^	*U*^13^	*U*^23^
N1	0.0432 (15)	0.0402 (14)	0.0416 (14)	−0.0020 (11)	−0.0024 (11)	−0.0081 (12)
N2	0.0399 (15)	0.0545 (16)	0.0540 (16)	−0.0090 (12)	0.0030 (12)	−0.0182 (14)
N3	0.0428 (15)	0.0531 (16)	0.0546 (16)	−0.0070 (13)	−0.0073 (13)	−0.0124 (13)
N4	0.0373 (15)	0.0555 (16)	0.0543 (16)	−0.0047 (12)	−0.0040 (12)	−0.0179 (14)
N5	0.0375 (14)	0.0680 (18)	0.0615 (18)	−0.0062 (12)	0.0024 (12)	−0.0322 (15)
N6	0.0405 (14)	0.0402 (14)	0.0443 (15)	−0.0105 (11)	−0.0026 (11)	−0.0117 (12)
N7	0.0405 (15)	0.0447 (15)	0.0468 (15)	−0.0047 (11)	−0.0044 (11)	−0.0107 (12)
N8	0.0414 (15)	0.0525 (16)	0.0564 (17)	−0.0053 (12)	0.0008 (12)	−0.0162 (13)
N9	0.0362 (14)	0.0584 (17)	0.0485 (15)	−0.0103 (12)	0.0028 (11)	−0.0141 (13)
N10	0.0401 (15)	0.0626 (17)	0.0674 (18)	−0.0084 (13)	−0.0063 (13)	−0.0296 (15)
O1	0.105 (2)	0.0779 (19)	0.0757 (19)	−0.0019 (17)	−0.0336 (17)	0.0054 (15)
O2	0.0770 (18)	0.0712 (17)	0.0722 (17)	−0.0083 (15)	0.0210 (14)	−0.0060 (14)
C1	0.0407 (18)	0.0519 (19)	0.0516 (19)	−0.0047 (15)	0.0015 (15)	−0.0127 (16)
C2	0.0346 (16)	0.0395 (16)	0.0397 (16)	−0.0041 (13)	−0.0008 (13)	−0.0086 (14)
C3	0.0356 (17)	0.0368 (16)	0.0401 (17)	−0.0022 (13)	−0.0043 (13)	0.0003 (13)
C4	0.0369 (18)	0.057 (2)	0.061 (2)	−0.0090 (15)	−0.0038 (16)	−0.0118 (18)
C5	0.0388 (17)	0.0430 (17)	0.0443 (18)	−0.0021 (13)	−0.0043 (14)	−0.0090 (15)
C6	0.066 (2)	0.0417 (18)	0.0365 (17)	0.0014 (15)	−0.0023 (15)	−0.0108 (14)
C7	0.072 (2)	0.0430 (18)	0.055 (2)	−0.0013 (16)	−0.0081 (17)	−0.0142 (16)
C8	0.071 (2)	0.050 (2)	0.057 (2)	0.0128 (19)	0.001 (2)	−0.0020 (18)
C9	0.082 (3)	0.136 (4)	0.094 (3)	0.003 (3)	0.021 (3)	0.046 (3)
C10	0.090 (3)	0.064 (2)	0.044 (2)	0.001 (2)	−0.0139 (18)	−0.0098 (17)
C11	0.076 (3)	0.072 (2)	0.061 (2)	0.007 (2)	0.0088 (19)	−0.025 (2)
C12	0.0390 (17)	0.0495 (18)	0.0470 (18)	−0.0084 (14)	−0.0061 (14)	−0.0085 (15)
C13	0.0333 (16)	0.0371 (16)	0.0392 (16)	−0.0083 (12)	0.0007 (12)	−0.0023 (13)
C14	0.0366 (17)	0.0387 (16)	0.0385 (16)	−0.0105 (13)	0.0033 (13)	−0.0043 (13)
C15	0.0388 (18)	0.061 (2)	0.053 (2)	−0.0028 (15)	0.0017 (15)	−0.0096 (17)
C16	0.0384 (17)	0.0437 (17)	0.0392 (17)	−0.0127 (14)	0.0050 (13)	−0.0078 (14)
C17	0.055 (2)	0.0488 (19)	0.0442 (18)	−0.0177 (15)	0.0027 (15)	−0.0142 (15)
C18	0.060 (2)	0.0439 (19)	0.063 (2)	−0.0120 (16)	0.0047 (17)	−0.0175 (17)
C19	0.054 (2)	0.051 (2)	0.056 (2)	−0.0205 (17)	−0.0019 (17)	−0.0063 (17)
C20	0.086 (3)	0.108 (3)	0.073 (3)	−0.002 (3)	−0.013 (2)	0.012 (3)
C21	0.083 (3)	0.074 (2)	0.055 (2)	−0.020 (2)	0.0121 (19)	−0.0231 (19)
C22	0.071 (2)	0.065 (2)	0.066 (2)	−0.0249 (19)	−0.0131 (19)	−0.0202 (19)

### Geometric parameters (Å, °)

**Table:**

N1—C1	1.364 (3)	C7—C8	1.495 (5)
N1—C3	1.368 (3)	C7—H7A	0.9700
N1—C6	1.491 (3)	C7—H7B	0.9700
N2—C1	1.307 (4)	C8—C9	1.488 (5)
N2—C2	1.383 (3)	C9—H9A	0.9600
N3—C4	1.320 (4)	C9—H9B	0.9600
N3—C3	1.353 (3)	C9—H9C	0.9600
N4—C4	1.334 (4)	C10—H10C	0.9600
N4—C5	1.356 (3)	C10—H10D	0.9600
N5—C5	1.333 (3)	C10—H10E	0.9600
N5—H5A	0.8600	C11—H11A	0.9600
N5—H5B	0.8600	C11—H11B	0.9600
N6—C12	1.364 (4)	C11—H11C	0.9600
N6—C14	1.382 (3)	C12—H12	0.9300
N6—C17	1.489 (3)	C13—C14	1.378 (4)
N7—C12	1.311 (3)	C13—C16	1.409 (4)
N7—C13	1.383 (3)	C15—H15	0.9300
N8—C15	1.331 (4)	C17—C21	1.520 (4)
N8—C14	1.339 (3)	C17—C22	1.522 (4)
N9—C15	1.340 (4)	C17—C18	1.530 (4)
N9—C16	1.341 (3)	C18—C19	1.502 (5)
N10—C16	1.332 (3)	C18—H18A	0.9700
N10—H10A	0.8600	C18—H18B	0.9700
N10—H10B	0.8600	C19—C20	1.491 (5)
O1—C8	1.205 (4)	C20—H20A	0.9600
O2—C19	1.205 (4)	C20—H20B	0.9600
C1—H1	0.9300	C20—H20C	0.9600
C2—C3	1.381 (4)	C21—H21A	0.9600
C2—C5	1.392 (4)	C21—H21B	0.9600
C4—H4	0.9300	C21—H21C	0.9600
C6—C11	1.514 (5)	C22—H22A	0.9600
C6—C10	1.519 (4)	C22—H22B	0.9600
C6—C7	1.534 (4)	C22—H22C	0.9600
			
C1—N1—C3	105.2 (2)	C6—C10—H10E	109.5
C1—N1—C6	127.4 (2)	H10C—C10—H10E	109.5
C3—N1—C6	127.1 (2)	H10D—C10—H10E	109.5
C1—N2—C2	104.0 (2)	C6—C11—H11A	109.5
C4—N3—C3	110.7 (2)	C6—C11—H11B	109.5
C4—N4—C5	117.3 (3)	H11A—C11—H11B	109.5
C5—N5—H5A	120.0	C6—C11—H11C	109.5
C5—N5—H5B	120.0	H11A—C11—H11C	109.5
H5A—N5—H5B	120.0	H11B—C11—H11C	109.5
C12—N6—C14	105.3 (2)	N7—C12—N6	114.8 (2)
C12—N6—C17	127.9 (2)	N7—C12—H12	122.6
C14—N6—C17	126.3 (2)	N6—C12—H12	122.6
C12—N7—C13	103.0 (2)	C14—C13—N7	111.5 (2)
C15—N8—C14	110.7 (2)	C14—C13—C16	117.1 (3)
C15—N9—C16	118.3 (2)	N7—C13—C16	131.2 (3)
C16—N10—H10A	120.0	N8—C14—C13	126.6 (3)
C16—N10—H10B	120.0	N8—C14—N6	127.9 (2)
H10A—N10—H10B	120.0	C13—C14—N6	105.5 (2)
N2—C1—N1	114.3 (3)	N8—C15—N9	129.4 (3)
N2—C1—H1	122.9	N8—C15—H15	115.3
N1—C1—H1	122.9	N9—C15—H15	115.3
C3—C2—N2	110.0 (2)	N10—C16—N9	119.0 (2)
C3—C2—C5	117.7 (3)	N10—C16—C13	123.3 (3)
N2—C2—C5	132.2 (2)	N9—C16—C13	117.7 (2)
N3—C3—N1	127.9 (3)	N6—C17—C21	108.4 (2)
N3—C3—C2	125.6 (3)	N6—C17—C22	110.1 (3)
N1—C3—C2	106.5 (2)	C21—C17—C22	108.8 (3)
N3—C4—N4	130.4 (3)	N6—C17—C18	108.0 (2)
N3—C4—H4	114.8	C21—C17—C18	109.6 (3)
N4—C4—H4	114.8	C22—C17—C18	111.8 (3)
N5—C5—N4	117.8 (3)	C19—C18—C17	117.3 (3)
N5—C5—C2	123.9 (3)	C19—C18—H18A	108.0
N4—C5—C2	118.2 (2)	C17—C18—H18A	108.0
N1—C6—C11	109.7 (2)	C19—C18—H18B	108.0
N1—C6—C10	107.7 (2)	C17—C18—H18B	108.0
C11—C6—C10	109.7 (3)	H18A—C18—H18B	107.2
N1—C6—C7	107.6 (2)	O2—C19—C20	119.9 (3)
C11—C6—C7	112.1 (3)	O2—C19—C18	123.5 (3)
C10—C6—C7	109.9 (3)	C20—C19—C18	116.6 (3)
C8—C7—C6	117.9 (3)	C19—C20—H20A	109.5
C8—C7—H7A	107.8	C19—C20—H20B	109.5
C6—C7—H7A	107.8	H20A—C20—H20B	109.5
C8—C7—H7B	107.8	C19—C20—H20C	109.5
C6—C7—H7B	107.8	H20A—C20—H20C	109.5
H7A—C7—H7B	107.2	H20B—C20—H20C	109.5
O1—C8—C9	121.4 (4)	C17—C21—H21A	109.5
O1—C8—C7	122.9 (3)	C17—C21—H21B	109.5
C9—C8—C7	115.7 (4)	H21A—C21—H21B	109.5
C8—C9—H9A	109.5	C17—C21—H21C	109.5
C8—C9—H9B	109.5	H21A—C21—H21C	109.5
H9A—C9—H9B	109.5	H21B—C21—H21C	109.5
C8—C9—H9C	109.5	C17—C22—H22A	109.5
H9A—C9—H9C	109.5	C17—C22—H22B	109.5
H9B—C9—H9C	109.5	H22A—C22—H22B	109.5
C6—C10—H10C	109.5	C17—C22—H22C	109.5
C6—C10—H10D	109.5	H22A—C22—H22C	109.5
H10C—C10—H10D	109.5	H22B—C22—H22C	109.5
			
C2—N2—C1—N1	0.2 (3)	C13—N7—C12—N6	−0.1 (3)
C3—N1—C1—N2	−0.3 (3)	C14—N6—C12—N7	0.3 (3)
C6—N1—C1—N2	−174.2 (3)	C17—N6—C12—N7	−171.4 (3)
C1—N2—C2—C3	0.0 (3)	C12—N7—C13—C14	−0.1 (3)
C1—N2—C2—C5	175.5 (3)	C12—N7—C13—C16	174.6 (3)
C4—N3—C3—N1	179.6 (3)	C15—N8—C14—C13	0.1 (4)
C4—N3—C3—C2	−1.0 (4)	C15—N8—C14—N6	178.7 (3)
C1—N1—C3—N3	179.7 (3)	N7—C13—C14—N8	179.1 (3)
C6—N1—C3—N3	−6.4 (5)	C16—C13—C14—N8	3.6 (4)
C1—N1—C3—C2	0.2 (3)	N7—C13—C14—N6	0.2 (3)
C6—N1—C3—C2	174.2 (2)	C16—C13—C14—N6	−175.2 (2)
N2—C2—C3—N3	−179.6 (3)	C12—N6—C14—N8	−179.1 (3)
C5—C2—C3—N3	4.2 (4)	C17—N6—C14—N8	−7.2 (5)
N2—C2—C3—N1	−0.1 (3)	C12—N6—C14—C13	−0.3 (3)
C5—C2—C3—N1	−176.4 (2)	C17—N6—C14—C13	171.6 (3)
C3—N3—C4—N4	−1.9 (5)	C14—N8—C15—N9	−2.5 (5)
C5—N4—C4—N3	1.4 (5)	C16—N9—C15—N8	0.8 (5)
C4—N4—C5—N5	−175.5 (3)	C15—N9—C16—N10	−174.7 (3)
C4—N4—C5—C2	2.1 (4)	C15—N9—C16—C13	3.2 (4)
C3—C2—C5—N5	172.9 (3)	C14—C13—C16—N10	172.7 (3)
N2—C2—C5—N5	−2.3 (5)	N7—C13—C16—N10	−1.7 (5)
C3—C2—C5—N4	−4.5 (4)	C14—C13—C16—N9	−5.2 (4)
N2—C2—C5—N4	−179.8 (3)	N7—C13—C16—N9	−179.6 (3)
C1—N1—C6—C11	−4.0 (4)	C12—N6—C17—C21	−126.0 (3)
C3—N1—C6—C11	−176.7 (3)	C14—N6—C17—C21	64.0 (4)
C1—N1—C6—C10	−123.4 (3)	C12—N6—C17—C22	−7.0 (4)
C3—N1—C6—C10	64.0 (4)	C14—N6—C17—C22	−177.1 (3)
C1—N1—C6—C7	118.2 (3)	C12—N6—C17—C18	115.3 (3)
C3—N1—C6—C7	−54.5 (4)	C14—N6—C17—C18	−54.7 (4)
N1—C6—C7—C8	−52.1 (4)	N6—C17—C18—C19	−56.0 (3)
C11—C6—C7—C8	68.6 (4)	C21—C17—C18—C19	−173.9 (3)
C10—C6—C7—C8	−169.1 (3)	C22—C17—C18—C19	65.3 (3)
C6—C7—C8—O1	−18.3 (5)	C17—C18—C19—O2	−8.0 (5)
C6—C7—C8—C9	163.2 (3)	C17—C18—C19—C20	172.5 (3)

### Hydrogen-bond geometry (Å, °)

**Table:**

*D*—H···*A*	*D*—H	H···*A*	*D*···*A*	*D*—H···*A*
N10—H10B···N4^i^	0.86	2.28	3.051 (3)	149
N10—H10A···N2^ii^	0.86	2.23	3.072 (3)	166
N5—H5B···N9^iii^	0.86	2.16	2.988 (3)	161
N5—H5A···N7^iv^	0.86	2.20	3.064 (3)	178

Symmetry codes: (i) *x*+1, *y*−1, *z*; (ii) *x*, *y*−1, *z*; (iii) *x*, *y*+1, *z*; (iv) *x*−1, *y*+1, *z*.
